# microRNA-34a inhibits epithelial mesenchymal transition in human cholangiocarcinoma by targeting Smad4 through transforming growth factor-beta/Smad pathway

**DOI:** 10.1186/s12885-015-1359-x

**Published:** 2015-06-16

**Authors:** Pengfei Qiao, Guodong Li, Wen Bi, Lianmeng Yang, Lei Yao, Dequan Wu

**Affiliations:** 1 Department of General Surgery, the Second Affiliated Hospital of Harbin Medical University, Harbin, 150086 People's Republic of China; 2 Department of General Surgery, the Fourth Affiliated Hospital of Harbin Medical University, Harbin, 150001 People's Republic of China; 3 Bio-Bank of Department of General Surgery, the Fourth Affiliated Hospital of Harbin Medical University, Harbin, 150001 People's Republic of China

**Keywords:** Cholangiocarcinoma, miR-34a, Smad4, Epithelial-mesenchymal transition, Transforming growth factor-beta

## Abstract

**Background:**

Extrahepatic Cholangiocarcinoma (EHCC) is one of the uncommon malignancies in the digestive system which is characterized by a poor prognosis. Aberrations of miRNAs have been shown involved in the progression of this disease. In this study, we evaluated the expression and effects of miR-34a on EHCC.

**Methods:**

miR-34a expression levels were detected in EHCC tissues, adjacent non-tumor tissues, normal bile duct (NBD) specimens of patients and cholangiocarcinoma (CC) cell lines by quantitative real-time polymerase chain reaction (qRT-PCR). Relationships between miR-34a with clinical characteristics of EHCC patients were further analyzed. Computational search, functional luciferase assay and western blot were further used to demonstrate the downstream target of miR-34a in CC cells. Immunohistochemistry was carried on to identify the downstream target gene of miR-34a in EHCC patients. Cell morphology, invasion and migration assays were further applied to confirm the anti-carcinogenic effects of miR-34a through the downstream target.

**Results:**

miR-34a expression was significantly decreased in human EHCC tissues and CC cell lines when compared with the adjacent non-tumor tissues and normal bile duct tissues. miR-34a was found correlated with the migration and invasion in EHCC patients. Smad4 was over-expressed in most of the EHCC patients and was further demonstrated as one of the downstream targets of miR-34a, which was involved in the progression of EHCC. Moreover, activation of miR-34a suppressed invasion and migration through TGF-beta/Smad4 signaling pathway by epithelial-mesenchymal transition (EMT) *in vitro*.

**Conclusions:**

Taken together, our results suggest that miR-34a inhibits invasion and migration by targeting Smad4 to suppress EMT through TGF- beta/Smad signaling pathway in human EHCC.

**Electronic supplementary material:**

The online version of this article (doi:10.1186/s12885-015-1359-x) contains supplementary material, which is available to authorized users.

## Background

Cholangiocarcinoma (CC) is a bile duct cancer, and is classified anatomically as intrahepatic CC (IHCC) or extrahepatic CC (EHCC). EHCC is a highly malignant cancer of the biliary tract [1, 2]. The incidence and mortality of EHCC is rising worldwide. Despite advances in surgical techniques, chemotherapies and radiotherapies, median survival of EHCC remains less than 24 months because of the patients are usually diagnosed at the advanced stage as the tumor has metastasized to regional lymph nodes or liver sites, which are the main prognostic factors in EHCC patients [3]. Exploring the molecular mechanisms underlying the initiation, progression, invasion and metastasis of EHCC is vital as it may provide new therapeutic targets, leading to improvements in the long-term survival of patients with EHCC.

MicroRNAs (miRNAs) are small noncoding RNAs of 20–22 nucleotides involved in the regulation of gene expression at a post-transcriptional level by binding to the target sites of messenger RNAs (mRNAs). miRNAs act as important post-transcriptional regulators of gene expression, and have recently emerged as key regulatory molecules in various cellular processes, including differentiation, self-renewal, proliferation and apoptosis [4]. It has been found that miRNAs regulate the expression of target genes by interacting with complementary sites in the 3'-untranslated region (UTR) of target mRNAs [5], and more than 30 % of human genes are regulated post transcriptionally by miRNAs [6]. miRNAs may function as oncogenes or tumor suppressors by targeting many cancer-associated genes in the progression of EHCC. The miR-34 family members share high sequence homology [7]. Among these, miR-34a is one of the earliest known tumor suppressors and is commonly deleted in various types of cancers. As a direct transcriptional target of p53, decreased expression of miR-34a is partly due to the mutations of p53 in tumors [8]. Recent research has found that down-regulation of miR-34a leads to a switch from Mnt (MAX network transcriptional repressor) to c-Myc expression during cholestatic cholangiocarcinogenesis in a mouse model [9]. Moreover, miR-34a can suppress tumor metastasis and invasion through a variety of signaling pathways in several cancers [10–14]. However, the anti-tumor function of miR-34a in EHCC is still not clear yet.

Transforming growth factor-β (TGF-β), which is a secreted homodimeric protein, belongs to a large family of pleiotropic factors that signal via heterotetrameric complexes of type I and type II serine/threonine kinase receptors. Important intracellular mediators of TGF-β signaling are members of the Smad family [15]. Smad4 is the common-mediator which cooperates with other transcription factors to regulate TGF-β signaling pathway [16]. The TGF-β signaling pathway has been shown to involve in various cellular responses in carcinogenesis of EHCC including cell proliferation and differentiation, migration and epithelial-mesenchymal transition (EMT) [17–19].

In the present study, miR-34a expression levels were detected in EHCC tissues, and CC cell lines. Relationships of miR-34a with clinical characteristics of EHCC patients were further examined. Moreover, Smad4 was demonstrated as a direct transcriptional target of miR-34a in CC. We identify miR-34a could mediate TGF-β/Smad4 signaling pathway induced EMT in the progression of cholangiocarcinoma.

## Methods

### Patients and tissue samples

EHCC tissues and adjacent non-tumor tissues used for qRT-PCR and/or immunohistochemistry (IHC) were collected from 27 EHCC patients who underwent potentially curative surgery between 2010 and 2011 at the Second Affiliated Hospital of Harbin Medical University (Harbin, China) and were verified by a pathologist. Seven primary normal bile duct (NBD) specimens were also collected from surgical resections performed for pancreatic cancer. These patients underwent a Whipple’s procedure. The hard and firm tumor tissues were trimmed free of normal tissue and snap frozen in liquid nitrogen immediately after resection. No patient in the current study received chemotherapy or radiation therapy before the surgery. The tumor stage was classified according to the 7th tumor-node-metastasis classification of the International Union against Cancer (UICC). All the patients signed informed consent forms according to our institutional guidelines, and the study was approved by Institutional Review Board (IRB) protocols of Harbin Medical University. Information on gender, age, stage of disease, and histological factors was extracted from medical records.

### Immunohistochemistry (IHC)

Immunohistochemical staining of sections for Smad4 expression was performed by a standard streptavidin-biotin peroxidase complex method [20]. Each 4-mm section was deparaffinised, rehydrated, and incubated with fresh 0.3 % hydrogen peroxide in methanol for 30 min at room temperature to block endogenous peroxidase activity. After rehydration through a graded series of ethanol solutions, the sections were autoclaved in 10 mM citrate buffer (pH 6.0) at 95 °C for 20 min and then cooled to 30 °C. After rinsing in 0.1 M phosphate buffer saline (PBS, pH 7.4), non-specific binding sites were blocked by incubation with 10 % normal rabbit serum for 30 min. The sections were then incubated with anti-Smad4 primary antibodies (Santa Cruz Biotechnology, USA) at a dilution of 1:100 in PBS containing 1 % bovine serum albumin at 4 °C overnight. The sections were washed in PBS, incubated with biotinylated anti-mouse IgG for 30 min at room temperature, and finally incubated in a streptavidin-biotin peroxidase complex solution (Nichirei Co., Tokyo, Japan). The chromogen, 3, 3′-diaminobenzidine tetra-hydrochloride, was applied as a 0.02 % solution containing 0.005 % H_2_O_2_ in 50 mM ammonium acetate-citrate acid buffer (pH 6.0). The sections were lightly counterstained with Mayer’s hematoxylin and mounted. Negative controls were established by replacing the primary antibody with normal rabbit serum. No detectable staining was evident in the negative controls.

### RNA extraction and quantitative real-time PCR (qRT-PCR)

qRT-PCR was used to confirm the expression levels of mRNAs and miRNAs. For mRNAs detection, total RNA from cultured cells and fresh surgical tissues was extracted using Trizol (Invitrogen, USA) according to the protocol. Reverse transcription was performed according to the protocol of High Capacity cDNA Reverse Transcription Kit (Applied Biosystems, USA). For miRNAs detection, total miRNA from cultured cells and fresh surgical tissues was extracted using the mirVana miRNA Isolation Kit (Ambion, USA), according to the manufacturer’s protocol. Complimentary DNA was synthesized from 2 μg of total RNA using the High Capacity cDNA Reverse Transcription Kit (Applied Biosystems, USA). The expression level of miRNA and mRNA were assessed with qRT-PCR using Power SYBR® Green (Applied Biosystems, USA) by an Applied Biosystems 7500 Sequence Detection system. The expression level of mRNA and miRNA was defined based on the threshold cycle (Ct), and relative expression levels were calculated using the 2^-∆∆Ct^ method, using the expression level of β-actin mRNA and U6 small nuclear RNA as a reference gene. The names of the genes and the primers are listed in Additional file 1: Table S1.

### Cell culture and quick transfection

The human EHCC cell lines QBC939 and HuCCT1 used in this study were purchased from American Type Culture Collection (Manassas, USA) and the human IHCC cell line RBE and HCCC9810 were obtained from the Cell Bank of the Chinese Academy of Sciences (Shanghai, China). Human intrahepatic biliary epithelial cells (HiBECs) were purchased from PriCells Biomedical Technology Co., Ltd. (Wuhan, China). All the cells were cultured according to the manufacturer’s instructions. A chemically modified antisense oligonucleotide and a synthetic miR-34a mimic (GenePharm Co. Ltd, China) were used to inhibit and increase miR-34a expression respectively. A scrambled oligonucleotide (GenePharm Co. Ltd, China) was used as a control. The transfections were performed using Lipofectamine TM 2000 transfection reagent (Invitrogen, USA) according to the manufacturer’s instructions. A mixture of Lipofectamine 2000 and RNA was added to CC cells, which were 70 % confluent, for 4–6 hrs, and the cells were then incubated for 24 hrs in fresh medium. After that, the cells were harvested using lysis buffer for luciferase assay. Total RNAs and protein were prepared 48 hrs after transfection and used for qRT-PCR or western blot analysis.

### Construction of promoter reporter plasmids and luciferase reporter assays

The fragment containing miR-34a binding sites in the Smad4 3′-UTR was amplified by PCR and inserted downstream of the firefly luciferase gene in a pGL3-promoter vector (Promega, Madison, WI, USA). The mutant reporter plasmids were constructed using the QuikChange mutagenesis kit (Stratagene, La Jolla, CA, USA). These constructed plasmids were all sequenced to confirm their orientation. Luciferase activity was measured with the Dual-Luciferase Reporter Assay System (Promega, Madison, WI, USA) as mentioned before [21, 22]. Promoter activities were expressed as the ratio between Firefly luciferase and Renilla luciferase activities.

### Western blot

Protein lysates were separated using 8 % or 10 % SDS-PAGE gel electrophoresis and transferred to nitrocellulose membranes (Amersham Pharmacia Biotech, USA). The membrane was probed with the following antibodies: anti-Smad4, anti-Snail, anti-E-cadherin, anti-N-cadherin (Santa Cruz Biotechnology, USA). Finally, the membrane was probed with Alexa Fluor® 680 donkey anti-mouse IgG (H + L) (1:5000) (Invitrogen, USA). Antibody binding was detected by Odyssey™ Infrared Imaging System (Li-Cor, Lincoln, NE). The names of the antibodies are listed in Additional file 2: Table S2.

### Cell migration and invasion assays

The invasive potential of cells was measured in 6.5 micrometers Transwell with 8.0 micrometers Pore Polycarbonate Membrane Insert (Corning, USA) according to the manufacturer’s instructions. The filter of top chamber was matrigel-coated with 50 μl of diluted matrigel following the standard procedure and incubated at 37 °C for 2 hrs. The lower chambers were filled with 600 μl of DMEM medium with or without TGF-β (5 ng/mL) (R&D Systems Inc., USA) containing 5 % FBS as chemoattractant for a further 24 hrs [19]. Cells were serum-free-starved overnight, and then harvested and resuspended in migration medium (DMEM medium with 0.5 % BSA). Then the suspension of 5,000 cells in 100 μl migration medium was added into each top chamber. After the cells were incubated for 16 hrs, the non-invading cells that remained on the upper surface were removed with a cotton swab. The invasive cells on the lower surface of the membrane insert were fixed with 4 % paraformaldehyde for 30 min, permeabilized with 0.2 % Triton X-100 at room temperature for 15 min, and then stained with 0.1 % crystal violet for 5 min. The number of cells on the lower surface, which had invaded through the membrane, was counted under a light microscope in five random fields at a magnification of 100×. The experiments were repeated three times independently and results were given as means ± SD. The procedure for transwell migration assays were the same as the transwell invasion assay except that the filter of top chamber was not coated with matrigel.

### Statistical analysis

All the presented data were expressed as the mean ± SD and representative results were from at least three independent experiments. Statistical comparisons were calculated by Student’s two-tailed *t*-test. When multiple comparisons were possible, ANOVA coupled with Tukey correction was used. Multivariate logistic regression analysis and Cox regression analysis were performed to analyze all factors in the Table 1 by backward variable selection. Survival curves for the patients were calculated using the Kaplan-Meier method, and analyzed using the Log-rank test. Correlation analysis between relative expressions of Smad4 and miR-34a was examined by logistic regression analysis. P < 0.05 was considered statistically significant. Statistical analysis was carried out using SPSS 21 (IBM Corporation Software Group, USA) or the GraphPad Prism 5.0 software package (GraphPad Software, Inc., USA).

**Table 1:** Relationship between miR-34a expression and clinicopathological features in EHCC patients

Clinicopathological features	n	miR-34a		*P* value
		Low	High	
Age (yr)				0.695
<60	14	4	10	
≥60	13	5	8	
Gender				1.000
Male	16	8	8	
Female	11	6	5	
Tumor size (cm)				0.449
<2	12	5	7	
≥2	15	9	6	
Pathological type				0.222
Adenocarcinoma	24	11	13	
Mucocellulare carcinoma	0	0	0	
Adenosquamous carcinoma	3	3	0	
Squamous carcinoma	0	0	0	
Undifferentiated carcinoma	0	0	0	
Cell differentiation				0.326
Well	4	3	1	
Moderately	8	5	3	
Poorly	15	8	7	
Bismuth classification				0.385
Bismuth I	7	1	6	
Bismuth II	13	10	3	
Bismuth III	6	2	4	
Bismuth IV	1	1	0	
Lymphatic node metastasis				0.004
Absent	9	1	8	
Present	18	13	5	
Clinical stages				<0.001
I + II	13	2	11	
III + IV	14	12	2	

*P* <0.05 is significant

## Results

### miR-34a expression in human EHCC tissues and CC cell lines, and the clinicopathological significance of miR-34a expression in EHCC patients

**Fig. 1:**
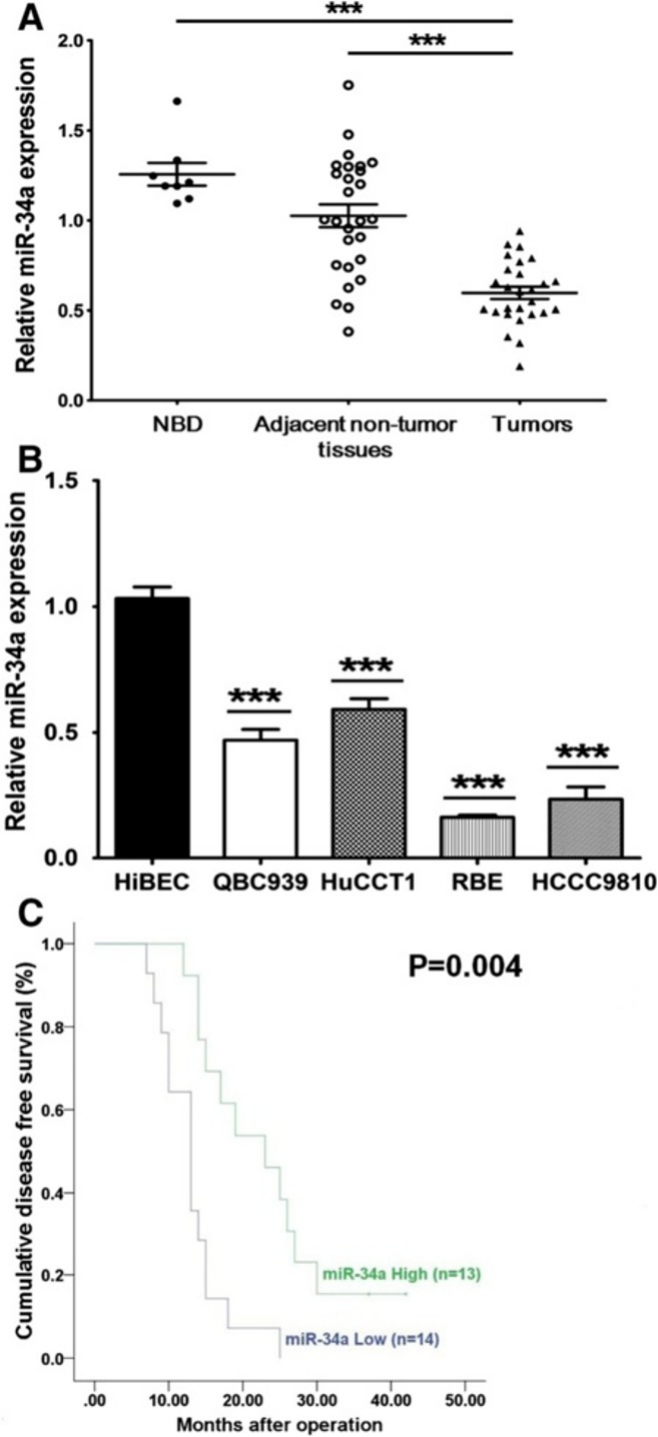
miR-34a expression in human EHCC tissues and CC cell lines, and the relationship between miR-34a expression and disease-free survival in EHCC patients. **a** The mRNA expression profile of miR-34a in 27 primary EHCC tissues compared to the adjacent non-tumor tissues and 7 normal bile duct tissues (NBD) determined by qRT-PCR (***P < 0.01). U6 was used as the internal control. **b** The mRNA expression level of miR-34a in 4 CC cell lines (QBC939, HuCCT1, RBE and HCCC9810) compared to HiBECs determined by qRT-PCR (***P < 0.01). U6 was used as the internal control. **c** miR-34a predicts disease-free survival in EHCC patients. The Kaplan-Meier curve of disease-free survival in patients with high miR-34a expression (n = 13) and low miR-34a expression (n = 14) (***P < 0.01). The median disease-free survival time was 13.07 months and 23.54 months in low- and high- miR-34a group, respectively (*P < 0.05)

The relative expression level of miR-34a in EHCC tissues was significantly lower than the NBD and the adjacent non-tumor tissues (P < 0.01, Fig. 1a). No significant difference was found between the NBD tissues and the adjacent non-tumor tissues. For further characterization of miR-34a expression in CC cell lines, HiBECs, EHCC cell line QBC939, HuCCT1 and the IHCC cell lines RBE and HCCC9810 were examined. qRT-PCR analysis revealed that the expression level of miR-34a was markedly decreased in all of the CC cell lines in comparison with the expression levels in HiBECs (P < 0.01, Fig. 1b). To further evaluate the clinical value of miR-34a in EHCC patients, we divided the patients into two groups according to the median value (5.113) of the expression level of miR-34a. The correlation between miR-34a and clinicopathological characteristics was then analyzed (Table 1). miR-34a showed lower expression levels in specimens with lymphatic metastasis (P = 0.004, Table 1) and in the advanced clinical stages (stage III, IV vs I, II) (P < 0.001, Table 1). The results of multivariate logistic regression analysis also showed that miR-34a expression related with clinical stages (P = 0.0013, Table 2). However, no association of miR-34a was observed with age, gender, tumor size, different pathological types, cell differentiation and Bismuth classification (P > 0.05, Table 1). Kaplan-Meier analysis showed that down-regulation of miR-34a was correlated with decreased disease-free survival (Fig. 1c, P = 0.004). Furthermore, Cox regression analysis was performed to analyze all the factors in Table 1 by backward variable selection. The results indicated that only miR-34a was selected into the model (P = 0.002). Thus, miR-34a was an independent prognostic indicator in EHCC.

**Table 2:** Multivariate logistic regression analysis for screening the influencial factors of miR-34a expression

Variable	b	OR (95 % CI)	*P* value
Intercept	−	−	0.0266
Clinical stages	−3.4965	0.030 (0.004 ~ 0.253)	0.0013
(I + II *versus* III + IV)			

Note: All clinicopathological features were employed for variable selection in the logistic regression analysis using a stepwise backward method. With the significance level for removal set at 0.05, only clinical stages was screened into the final model

### miR-34a and Smad4 protein levels are inversely expressed in human EHCC tissues

**Fig. 2:**
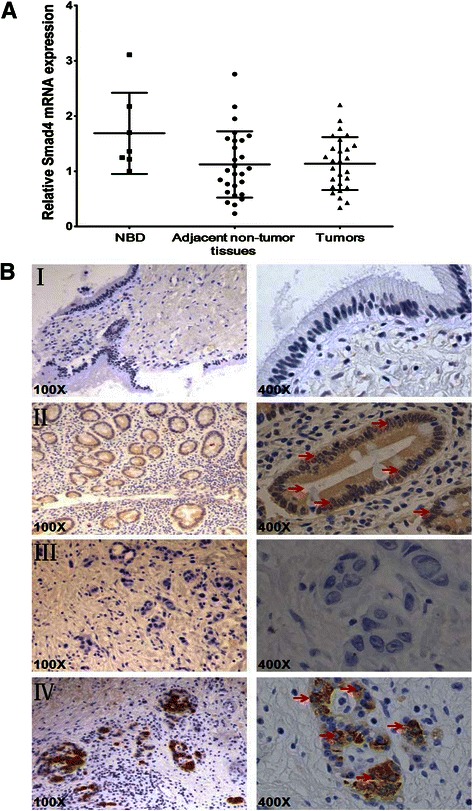
The expression levels of Smad4 in the primary EHCC and NBD specimens. **a** The mRNA expression level of Smad4 in the primary EHCC, the adjacent non-tumor tissues and NBD specimens determined by qRT-PCR. U6 was used as the internal control. **b** Immunohistochemical staining of Smad4 protein expression in primary EHCC and NBD samples. The arrows indicated nuclear staining of Smad4 in both NBD and EHCC samples. (*I*) Low Smad4 protein expression in a NBD. (*II*) High Smad4 protein expression in a NBD. (*III*) Reduced Smad4 protein expression in a primary EHCC. (*IV*) High Smad4 protein expression in a primary EHCC. Original magnification, 100× and 400× respectively for each slide

 In order to determine the clinical significance of miR-34a target genes in EHCC, Sanger miRNA database (http://www.mirbase.org/) and Targetscan (http://www.targetscan.org/) were used to predict the candidates of miR-34a. Moreover, both miR-34a and TGF-β/Smad4 pathway have been shown to involve in mediating metastasis and invasion in various types of cancers including cholangiocarcinoma [23, 24]. Thus, we examined Smad4 expression in the human clinical specimens. It was found that the mRNA level of Smad4 was not pronouncedly inhibited in human EHCC tissues compared with NBD and adjacent non-tumor tissues (Fig. 2a). The expression of Smad4 protein levels were further detected in 27 EHCC and 7 NBD specimens by IHC. As shown in Fig. 2b, positive staining of Smad4 was mainly identified in the cell cytoplasm of cancer cells in 18 of 27 tumor tissues and negative staining for Smad4 protein in 5 of 7 NBD tissues. Even a down-regulation of miR-34a with negative staining for Smad4 proteins were found in 7 patients, low expression of miR-34a is associated with positive staining for Smad4 protein in 18 EHCC samples. Logistic regression analysis was performed to determine a negative correlation between Smad4 protein expression level and miR-34a level (B = −3.035, P = 0.01) among the total 27 EHCC tissues. These data suggest that miR-34a expression is inversely correlated with Smad4 in EHCC patients, and miR-34a might play a critical role on Smad4 regulation in over a half but not all of the EHCC patients.

### miR-34a directly targets Smad4 in CC cells

**Fig. 3:**
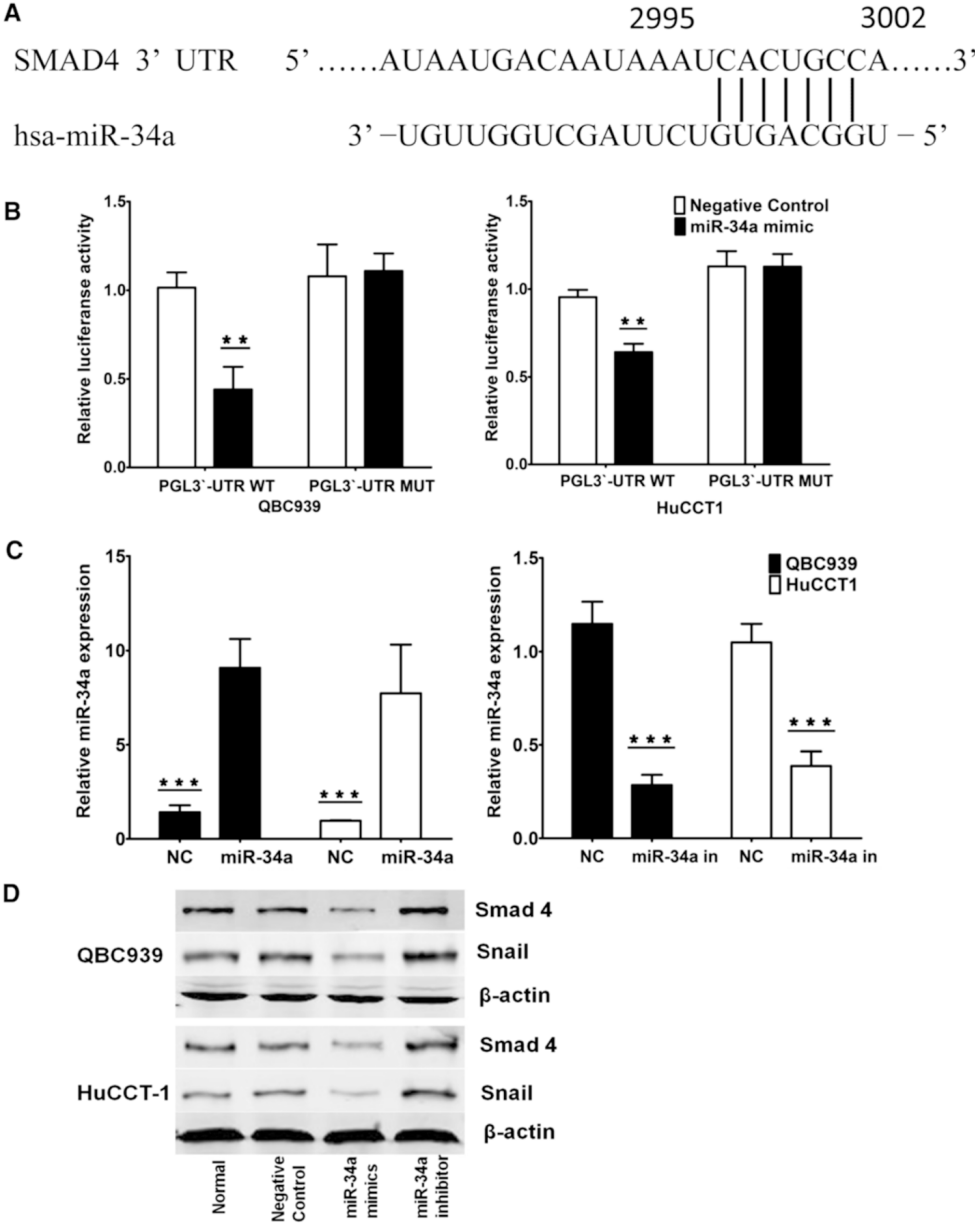
Smad4 is a direct miR-34a target. **a** miRNA target prediction screened one computative miR-34a binding site at Smad4-three prime untranslated region (3′-UTR). **b** 3′-UTR luciferase reporter assay showed a reduction of relative luciferase activity of wild-type Smad4 3′-UTR by pre-miR-34a in QBC939 and HuCCT1 cells (**P < 0.05). **c** qRT-PCR analysis of expression of miR-34a treated with miR-34a mimics or miR-34a inhibitor in QBC939 and HuCCT1 cells (***P < 0.01). U6 was used as a loading control. Error bars represent mean ± SD from three independent experiments. **d** Western blot analysis of Smad4 and Snail expression treated with miR-34a inhibitor or mimic in QBC939 and HuCCT1 cells. β-actin levels were used as internal loading control

Based on the Sanger miRNA database and TargetScan software, one potential binding site of miR-34a in the 3′-UTR of Smad4 (from 2995 to 3002) was predicted (Fig. 3a). To test the specific regulation through the predicted binding site, we constructed a reporter vector consisting of the luciferase coding sequence followed by the 3′-UTR of Smad4. A dual luciferase reporter assay was performed in QBC939 and HuCCT1 cell lines. As shown in Fig. 3b, a significant decrease in relative luciferase activity was observed when pGL3-Smad4-3′-UTR was cotransfected with a miR-34a mimic compared with the vector-only control. Moreover, partial mutation of the perfectly complementary sites in the 3′-UTR of Smad4 abolished the suppressive effect due to the disruption of the interaction between miR-34a and Smad4 (Fig. 3b).

Furthermore, to investigate the biological function of miR-34a in CC cells, we transfected miR-34a mimic oligonucleotides or miR-34a inhibitor oligonucleotides into the EHCC cell lines (QBC939 and HuCCT1) to further increase or decrease the endogenous level of miR-34a (Fig. 3b). The protein expression levels of Smad4 and Snail, which are downstream targets of TGF-β/Smad4 pathway, were found decreased by transfection with the miR-34a mimics but increased by transfection with a miR-34a inhibitor in both QBC939 and HuCCT1 cells (Fig. 3d). However, Smad4 mRNA levels were not significantly influenced by the over-expression or inhibition of miR-34a *in vitro* (Additional file 3: Figure S1). These data suggest that Smad4 expression was primarily inhibited by miR-34a at the translational level. Together, these results confirmed that Smad4 is a direct target of miR-34a and is regulated by miR-34a in CC cell lines.

### Up-regulation of miR-34a represses the EMT via TGF-β/Smad signaling pathway in CC cell lines

**Fig. 4:**
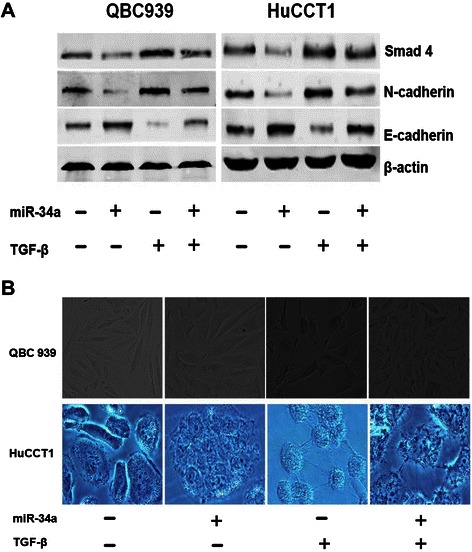
miR-34a antagonizes Smad4-mediated TGF-β induction of EMT *in vitro.***a** Both of the QBC939 and HuCCT1 cells were transfected with miR-34a mimics or scramble oligos, and treated with or without TGF-β simultaneously. After 48 hrs, cells lysate were examined with indicated antibodies by Western blot. β-actin was used as loading control. **b** Morphological investigations of the EHCC cells. Both QBC939 and HuCCT1 cells were transfected with miR-34a mimics then treated with or without 5 ng/ml of TGF-β for 24 hrs. Cells transfected with miR-34a scramble oligos were used as the controls (original magnification: ×200)

As Smad4 is the common-smad protein for the transduction of TGF-β signaling pathway, which plays important roles through EMT in carcinogenesis [16], the repression of Smad4 by miR-34a may impair this signaling pathway in EHCC. To further investigate the role of miR-34a in the progression of EHCC by its ability to repress EMT, we examined the effects of miR-34a on the downstream targets of TGF-β/Smad4 pathway in both QBC939 and HuCCT1 cells. The cells were transfected with miR-34a mimics or scramble oligos, and simultaneously treated with TGF-β. Western blot analysis showed that compared with TGF-β treatment alone, transfection of miR-34a mimics increased E-cadherin expression levels while decreasing Smad4 and N-cadherin protein levels (Fig. 4a). The morphological changes of EHCC cells were detected after transfected with miR-34a mimics and/or treated with TGF-β. The results showed that, after transfected with miR-34a mimics, the EHCC cells displayed a cobblestone-like morphology, and cell-to-cell adhesion was more intact compared with the control cells. However, when the cells were treated with TGF-β, a spindle-shaped morphology was developed, the cell-to-cell adhesions became weak, and the cells were scattered. Interestingly, after treated with both miR-34a mimics and TGF-β, the cells were assembled closely compared with miR-34a mimic transfection group (Fig. 4b). These data suggest that miR-34a could antagonize Smad4-mediated TGF-β induction of EMT *in vitro*.

### miR-34a suppresses the activation of TGF-β/Smad4 signal-induced invasion and migration in CC cell lines

**Fig. 5:**
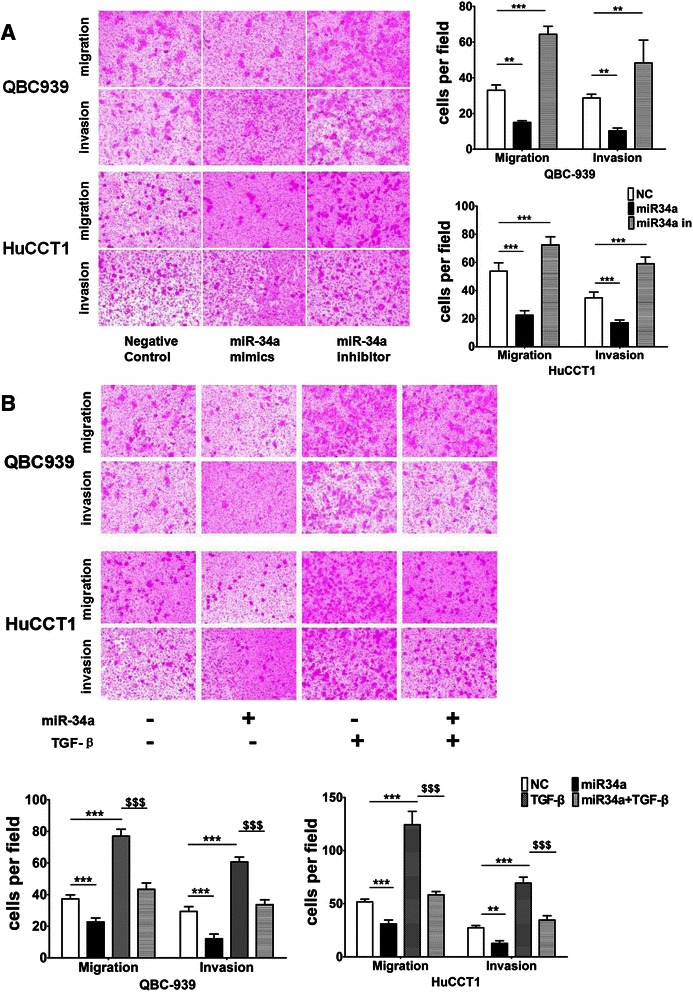
miR-34a suppresses EHCC cells invasion and migration through TGF-β/Smad4 signaling pathway. **a** The migration and invasive properties of EHCC cells treated with the empty vector, miR-34a mimic or miR-34a inhibitor were analyzed using a cell invasion assay in transwell chambers. Representative images of cells that had migrated into the lower chamber are shown (left panel, original magnification: ×100), and quantitative data are also presented (right panel, **P < 0.05 and ***P < 0.01). The average numbers of cells per field of view from three different experiments are plotted. **b** Both QBC939 and HuCCT1 cells were transfected with miR-34a mimics then treated with or without 5 ng/ml of TGF-β for 24 hrs to evaluate cell invasion and migration activities. Cells transfected with miR-34a scramble oligos were used as the controls. Representative images of cells in the lower section of a transwell chamber are shown to demonstrate the migration and invasive properties of QBC939 and HuCCT1 cells when transfected with the empty vector, miR-34a mimic, TGF-β or a mixed miR-34a and TGF-β (left panel). Quantitative analyses of the migrated and invasion cells are also shown (right panel, **P < 0.05 and ***P < 0.01 indicates miR-34a mimic, TGF-β or a mixed miR-34a and TGF-β *vs.* NC. $$$P < 0.01 means a mixed miR-34a and TGF-β *vs.* TGF-β). Data are plotted as the average number of cells per field of view from three different experiments (original magnification: ×100)

EMT is often linked to a gain in the migratory and invasive properties of cells [19]. To further investigate whether miR-34a suppresses cell invasion and migration through TGF-β/Smad4 signaling pathway in EHCC, both QBC939 and HuCCT1 cells were transfected with miR-34a mimics then treated with or without TGF-β. Transwell migration and invasion assays were then performed after transfection. It was found that transfection with miR-34a significantly suppressed cell migration in both QBC939 and HuCCT1 cells. Similarly, invasion capacity was also significantly down-regulated in both of the cell lines (Fig. 5a). The cell migration and in invasion capacities were all induced in both QBC939 and HuCCT1 cell lines after treated with TGF-β (Fig. 5b). However, these inductions were inhibited by the treatment with miR-34a mimics in both QBC939 and HuCCT1 cells (Fig. 5b). Moreover, Snail protein expression level was decreased by transfection with miR-34a mimics but increased by transfection with miR-34a inhibitor in both QBC939 and HuCCT1 cells (Fig. 3d). Snail is one of the specific downstream targets of TGF-β/Smad4 in regulation of EMT [25]. These results indicate that miR-34a participates in the regulation of cell migration and invasion in EHCC cells through down-regulation of TGF-β/Smad4 signaling pathway.

## Discussion

miR-34a is one of the most prominent miRNAs implicated in the development and progression of human cancers [7]. It is down-regulated in many human cancers including hepatocellular, ovarian, prostate and urothelial carcinoma, as well as colon, head and neck, gastric, breast, lung and pancreatic cancers [8, 10–14, 26–29]. In the present study, we found that miR-34a expression was significantly decreased in human EHCC tissues and CC cell lines when compared with the adjacent non-tumor tissues, NBD tissues and the HiBECs. Smad4 is further demonstrated as one of the targets of miR-34a, which was involved in the migration and invasion in EHCC cells. We also found activation of miR-34a suppresses the invasion and migration through TGF-β/Smad4 signaling pathway *in vitro*.

Previous studies have provided evidence for the important roles of deregulated expression of miRNAs in the pathogenesis of CC, including miR-29, miR-122, miR-124, miR-145, miR-146a, miR-200c, miR-370, miR-373, miR-376c and miR-494 [30–36]. Some reports have demonstrated that miR-34a to be an important anti-oncogene by regulation of different downstream targets in various types of cancers [7, 8, 10–14, 26–29]. Down-regulation of miR-34a has been found in left and median bile duct ligation (LMBDL) mouse livers [9]. However, the role of miR-34a in EHCC has yet to be elucidated. In the present study, we showed that the expression of miR-34a was down-regulated in EHCC tissues. These data are consistent with the decreased expression levels of miR-34a in the digestive system cancers which has been demonstrated by several groups [9, 26–29]. miR-34a functions as a tumor-suppressor miRNA, and can target many downstream genes in the development and progression of carcinogenesis. Our clinical analysis showed that down-regulation of miR-34a is correlated with lymphatic metastasis and advanced clinical stages. Moreover, lower expression of miR-34a correlated with the decreased disease-free survival in these EHCC patients. These data suggest that miR-34a is more likely involved in the metastasis or invasion during the progression of EHCC. Recent studies have found that miR-34a represses RhoA, a regulator of cell migration and invasion, by suppressing c-Myc–Skp2–Miz1 transcriptional complex that activates RhoA in human prostate cancer cells [37]. miR-34a was also found to reduce cell proliferation and invasiveness partially through its inhibitory effect on Delta-like 1 (DLL1) in choriocarcinoma [11]. Thus, miR-34a could also regulate some downstream targets in the progress of metastasis or invasion of EHCC.

Since miRNAs are generally involved in the pathogenesis of cancer by directly regulating the expression of their targets at a post-transcriptional level, we applied bioinformatic methods to predict the potential targets of miR-34a. Further investigation showed that miR-34a suppresses the activity of a luciferase reporter gene fused with the 3′-UTR of Smad4 mRNA, which is dependent on the miR-34a binding sequence. Our data revealed that miR-34a directly targets the 3′-UTR of Smad4, and that ectopic expression of miR-34a represses Smad4 protein level in CC cell lines. Interestingly, recent report suggested that miR-34a plays a critical role in the progression of cardiac fibrosis by increasing Smad4 expression to activate TGF-β1 [38]. These controversial results may due to the different experimental models and/or the various functions of miR-34a in different diseases. Besides, over-expression of miR-34a also inhibited the expression of TGF-β/Smad4 downstream targets N-cadherin and induced the expression level of E-cadherin. N-cadherin and E-cadherin are highly involved in the EMT during carcinogenesis [20]. EMT is a complex process in which epithelial cells acquire the characteristics of invasive mesenchymal cells. EMT has been implicated in cancer progression and metastasis. Several oncogenic pathways such as TGF-β, Wnt, and Notch signaling pathways have been shown to induce EMT [39]. The role of TGF-β in EMT, tumor invasiveness and metastasis has been firmly established *in vitro* and *in vivo* studies [28, 40], including in human EHCC. More importantly, TGF-β-induced activation of Smad complexes has been shown to play a crucial role during the induction of EMT [19]. Several reports have also shown that the levels of transcription factors driving EMT are controlled by miRNAs including miR-34a [26, 41–44]. Thus, our data showed that activation of miR-34a could antagonize Smad4-mediated TGF-β induction of EMT process through regulation of E-cadherin and N-cadherin expression. Snail, which is a downstream target of TGF-β/Smad4 signaling pathway, was also decreased by increasing miR-34a expression but increased by using miR-34a inhibitor in EHCC cells.

Moreover, the expression levels of miR-34a and Smad4 are inversely correlated in human clinical specimens of EHCC. Although there are a few samples with both miR-34a down-regulation and negative staining for Smad4 proteins by IHC, the protein level of Smad4 was increased in most of our EHCC tissues compared with NBD tissues. For those EHCC specimens, which did not have inverse correlation of miR-34a and Smad4 expression, we speculate that other factors might antagonize or interfere with the effect of miR-34a on Smad4. The expression pattern of individual miRs with strict tissues, the clinical-feature-specificity or the different target genes involved in the unique regulation network of EHCC may all involved in the effect of miR-34a on Smad4 [35, 45]. These speculations need further investigations in the future. Our results showed forced up-regulation of miR-34a significantly inhibited the protein expression of Smad4, and inhibited CC cells invasion and migration. The contrast results were observed when the CC cells were treated with the miR-34a inhibitor. These results identified Smad4 as a novel target of miR-34a in the EMT process of EHCC. Based on the contrasting expression patterns of miR-34a and Smad4 in most of the EHCC tissues and our *in vitro* data, we proposed that miR-34a is involved in the pathogenesis of EHCC by directly inhibiting the protein expression of Smad4. Therefore, our studies demonstrated that miR-34a functions as a tumor-suppressor miRNA by inhibiting TGF-β/Smad-induced EMT in CC cells.

## Conclusion

In summary, our results have identified miR-34a as a tumor suppressive miRNA in human EHCC, which acts at least in part through the repression of Smad4. Decreased expression of miR-34a in EHCC patients is correlated with lymphatic metastasis, advanced clinical stages and overall survival rate. Loss of the miR-34a expression leads to an induction of Smad4 and activation of TGF-β/Smad4 signaling pathway, which accelerate CC cells invasion and migration via EMT. Taken together, our data provide new insights into the potential contribution of miR-34a in inhibition the progression of EHCC, and suggest miR-34a is a useful molecule target for developing new therapeutic method against EHCC.

## Additional files

Additional file 1: Table S1. Primers used for qRT-PCR.
Additional file 2: Table S2. Antibodies used for Western Blot.
Additional file 3: Figure S1. Smad4 mRNA levels were not significantly influenced by the over-expression or inhibition of miR-34a *in vitro.* qRT-PCR analysis of Smad4 mRNA expression levels treated with miR-34a inhibitor or mimic in QBC939 and HuCCT1 cells. β-actin levels were used as internal loading control.

## Abbreviations

UTR: 3′-untranslated region
CC: Cholangiocarcinoma
DMEM: Dulbelcco’s Modified Eagle Media
EMT: Epithelial-mesenchymal transition
EHCC: Extrahepatic CC
FBS: Fetal bovine serum
HiBECs: Human intrahepatic biliary epithelial cells
UICC: International Union against Cancer
IHCC: Intrahepatic CC
IHC: Immunohistochemistry
miRNA: Micro RNA
Mnt: MAX network transcriptional repressor
NBD: Normal bile duct
PBS: Phosphate buffer saline
qRT-PCR: Quantitative real time Reverse Transcription-PCR
TGF-β: Transforming growth factor-β
