# Supplementary material for: microRNA-34a inhibits epithelial mesenchymal transition in human cholangiocarcinoma by targeting Smad4 through transforming growth factor-beta/Smad pathway
Source: BMC Cancer. 2015 Jun 16;15:469. doi: 10.1186/s12885-015-1359-x (PMC4477414; doi:10.1186/s12885-015-1359-x)
Supplement: Additional file 1: Table S1. — Primers used for qRT-PCR. [file 12885_2015_1359_MOESM1_ESM.doc]

| **Supplemental Table 1: Primers used for qRT-PCR** | |
| --- | --- |
| **Primer Name** | **Primer Sequence: 5'-3'** |
| **Forward Smad4** | **AGGATCAGTAGGTGGAATAG** |
| **Reverse Smad4** | **TCTAAAGGTTGTGGGTCTGC** |
| **Forward β-actin** | **ATGTTGAGACCTTCAACACC** |
| **Reverse β-actin** | **AGGTAGTCAGTCAGGTCCCGGCC** |
| **Forward miR-34a** | **CCTCCTGCATCCTTTCTTT** |
| **Reverse miR-34a** | **CCTGTGCCTTTTTCCTTCC** |
| **Forward U6** | **CTCGCTTCGGCAGCACA** |
| **Reverse U6** | **AACGCTTCACGAATTTGCGT** |
|  |  |
|  |  |
|  |  |
|  |  |
|  |  |
|  |  |
|  |  |
|  |  |
|  |  |
|  |  |
|  |  |
|  |  |
|  |  |
|  |  |
|  |  |
|  |  |
|  |  |
|  |  |
|  |  |
|  |  |
|  |  |
|  |  |
|  |  |
|  |  |
