# Supplementary material for: microRNA-34a inhibits epithelial mesenchymal transition in human cholangiocarcinoma by targeting Smad4 through transforming growth factor-beta/Smad pathway
Source: BMC Cancer. 2015 Jun 16;15:469. doi: 10.1186/s12885-015-1359-x (PMC4477414; doi:10.1186/s12885-015-1359-x)
Supplement: Additional file 2: Table S2. — Antibodies used for Western Blot. [file 12885_2015_1359_MOESM2_ESM.doc]

| **Supplemental Table 2: Antibodies used for Western Blot** | | | |  | |  |
| --- | --- | --- | --- | --- | --- | --- |
| **Name of Antibody** | **Manufacturer** | **Species** | **Dilution** | |  | |
| **Smad4** | **sc-7966 (Santa Cruz Biotechnology, USA)** | **Mouse** | **1:500** | |  | |
| **Snail** | **Sc-271977 (Santa Cruz Biotechnology, USA)** | **Mouse** | **1:500** | |  | |
| **E-cadherin** | **sc-59778 (Santa Cruz Biotechnology, USA)** | **Mouse** | **1:200** | |  | |
| **N-cadherin** | **sc-59987 (Santa Cruz Biotechnology, USA)** | **Mouse** | **1:500** | |  | |
| **β-actin** | **sc-69879 (Santa Cruz Biotechnology, USA)** | **Mouse** | **1:1000** | |  | |
|  |  |  |  | |  | |
|  |  |  |  | |  | |
|  |  |  |  | |  | |
|  |  |  |  | |  | |
|  |  |  |  | |  | |
|  |  |  |  | |  | |
|  |  |  |  | |  | |
|  |  |  |  | |  | |
|  |  |  |  | |  | |
|  |  |  |  | |  | |
