# Supplementary figures and images for: microRNA-34a inhibits epithelial mesenchymal transition in human cholangiocarcinoma by targeting Smad4 through transforming growth factor-beta/Smad pathway
Source: BMC Cancer. 2015 Jun 16;15:469. doi: 10.1186/s12885-015-1359-x (PMC4477414; doi:10.1186/s12885-015-1359-x)

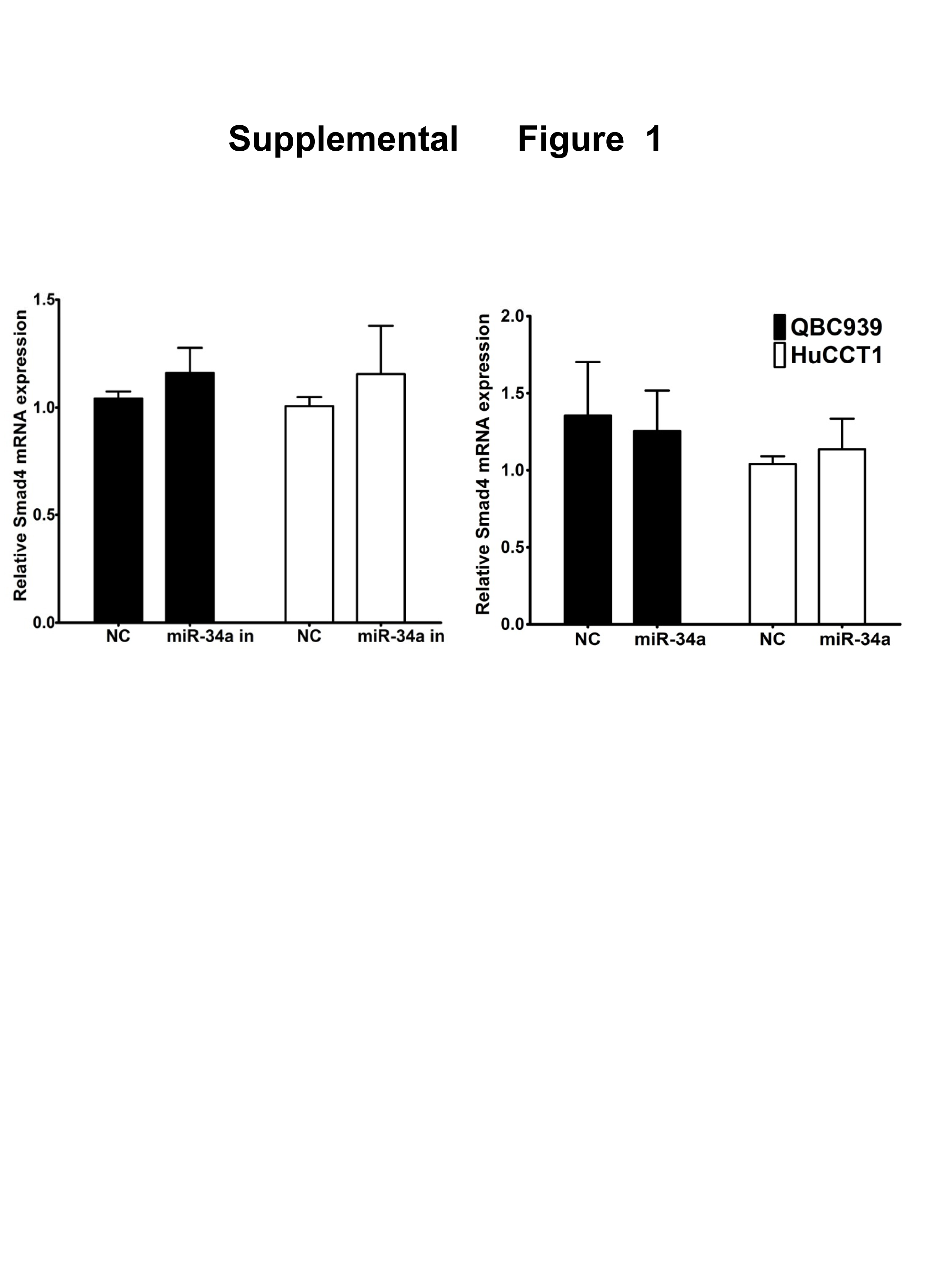

Supplement: Additional file 3: Figure S1. — Smad4 mRNA levels were not significantly influenced by the over-expression or inhibition of miR-34a in vitro. qRT-PCR analysis of Smad4 mRNA expression levels treated with miR-34a inhibitor or mimic in QBC939 and HuCCT1 cells. β-actin levels were used as internal loading control. [file 12885_2015_1359_MOESM3_ESM.tiff]
